# Assessment of Peripheral Nervous System Alterations in Patients with the Fabry Related *GLA*-Variant p.A143T

**DOI:** 10.3390/diagnostics10121027

**Published:** 2020-11-30

**Authors:** Tim Godel, Katharina von Cossel, Reinhard E. Friedrich, Markus Glatzel, Sima Canaan-Kühl, Thomas Duning, Moritz Kronlage, Sabine Heiland, Martin Bendszus, Nicole Muschol, Victor-Felix Mautner

**Affiliations:** 1 Department of Neuroradiology, Neurological University Clinic, Heidelberg University Hospital, Im Neuenheimer Feld 400, 69120 Heidelberg, Germany; moritz.kronlage@med.uni-heidelberg.de (M.K.); sabine.heiland@med.uni-heidelberg.de (S.H.); martin.bendszus@med.uni-heidelberg.de (M.B.); 2 Department of Pediatrics, University Medical Center Hamburg-Eppendorf, Martinistraße 52, 20246 Hamburg, Germany; k.cossel@uke.de (K.v.C.); muschol@uke.de (N.M.); 3 Department of Oral and Maxillofacial Surgery, University Medical Center Hamburg-Eppendorf, Martinistraße 52, 20246 Hamburg, Germany; r.friedrich@uke.de; 4 Institute of Neuropathology, University Medical Center Hamburg-Eppendorf, Martinistraße 52, 20246 Hamburg, Germany; m.glatzel@uke.de; 5 Division of Nephrology and Intensive Care Medicine, CCM, Charité Universitätsmedizin Berlin, Charitéplatz 1, 10117 Berlin, Germany; sima.canaan@charite.de; 6 Department of Neurology, University Hospital of Münster, Albert-Schweitzer-Campus 1, 48149 Münster, Germany; Thomas.Duning@ukmuenster.de; 7 Department of Neurology, University Medical Center Hamburg-Eppendorf, Martinistraße 52, 20246 Hamburg, Germany; v.mautner@uke.de

**Keywords:** Magnetic Resonance Neurography, dorsal root ganglia, neuropathic pain, peripheral neuropathy, Fabry disease

## Abstract

The purpose of this study is to examine alterations of the peripheral nervous system (PNS) in oligo-symptomatic patients carrying the Fabry related *GLA*-gene variant p.A143T by Magnetic Resonance Neurography (MRN) and skin biopsy. This prospective study assessed dorsal root ganglia (DRG) volume L3 to S2, vascular permeability of the DRG L5, S1, and the spinal nerve L5 in five patients carrying p.A143T in comparison to patients with classical Fabry mutations and healthy controls. Moreover, skin punch biopsies above the lateral malleolus of the right foot were obtained in four patients and intraepidermal nerve fiber density (IENFD) was counted individually. Compared to controls, DRG volumes of p.A143T patients were enlarged by 30% (L3, *p* < 0.05), 35% (L4, *p* < 0.05), 29% (L5, *p* = 0.15), 36% (S1, *p* < 0.01), and 18% (S2, *p* < 0.05), but less pronounced compared to patients carrying a classical Fabry mutation. Compared to healthy controls, vascular permeability was decreased by 40% (L5 right), 49% (L5 left), 48% (S1 right), and 49% (S1) (*p* < 0.01–*p* < 0.001), but non-significant less than patients carrying a classical Fabry mutation. Compared to sex-matched 5% lower normative reference values per decade, IENFD was decreased in three of four patients. MRN and determination of IENFD is able to detect early alteration of the PNS segment in oligo-symptomatic patients with the disease-modifying *GLA*-variant p.A143T on an individual basis. This procedure might also help in further *GLA*-variants of uncertain significance for early identification of patients with single major organ manifestation.

## 1. Introduction

Fabry disease (FD) is a life-limiting, multi-organ lysosomal storage disorder with painful small fiber neuropathy (SFN) as one of its earliest clinical presentations [1,2]. Mutations in the alpha-galactosidase A (GLA) gene cause a deficiency of the enzyme alpha-GLA and a subsequent intracellular accumulation of glycolipids, mainly globotriaosylceramide (Gb3), in the cardiovascular system, the renal system, and the central and peripheral nervous system (PNS) [3]. Neuropathic pain and pain attacks are commonly reported early symptoms in FD, reflecting damage to the small fiber neurons of the peripheral nervous system [4,5]. To promote early diagnosis and to prevent irreversible long-term complications, a better understanding of the underlying pathophysiology of these early painful symptoms is of high clinical relevance. By applying high-resolution peripheral nerve imaging (MR-Neurography, MRN) in patients with classical Fabry mutations, we described the fundamental role of the dorsal root ganglia (DRG) in the development of a painful SFN as an early and predominant symptom of major organ involvement [6,7]. More than 900 pathogenic mutations in the *GLA*-gene have been described so far and were subsequently divided into disease-causing mutations and disease modifying variants [8,9]. While there is an agreement on diagnostic criteria and treatment of multi-symptomatic patients carrying a classical, disease-causing mutation in general, the therapeutic strategies in oligo-symptomatic patients with a disease-modifying variant are still under debate [10]. While some authors recommend no specific diagnostic monitoring and treatment for these patients, there are other reports of oligo-symptomatic patients with single clinical, laboratory, and/or imaging features that are partly reminiscent to those seen in classical, multi-symptomatic Fabry patients, indicating a potential therapeutic consequence [11,12,13,14,15,16]. In a recent study in a group of oligo-symptomatic patients carrying the p.D313Y variant, we were able to show by MRN that these patients reveal pathological PNS features, which are commonly seen in multi-symptomatic Fabry patients with classical mutations [17]. To assess early PNS alterations also on an individual basis, we here investigated oligo-symptomatic patients with the high-prevalent *GLA*-gene variant p.A143T by morphometric and functional MRN and skin biopsy.

## 2. Patients and Methods

### 2.1. Clinical and Demographic Patient Data

The study was performed in accordance with the Declaration of Helsinki, approved by the institutional ethics board (S398-2012) and written informed consent was obtained from all patients. Skin biopsies were performed as a standard of care procedure in the work up of *GLA*-variants of unknown significance. As biopsy findings were acquired during clinical routine visits and patient data were anonymized before analysis, an additional approval for reporting biopsy results was not required (“Ethikkommission der Ärztekammer“ Germany (§ 9 Abs. 2, Hamburgisches Kammergesetz für die Heilberufe; § 15 Abs. 1, Berufsordnung für Hamburger Ärzte und Ärztinnen)).

Overall, we included five patients (two male, three female, mean age 58.40 years, range 37–71 years) carrying the Fabry related *GLA*-gene variant p.A143T and no additional classical Fabry mutation, as confirmed by genetic testing. Moreover, the Mainz severity score index (MSSI) [18] and lyso-Gb3 level were determined in all patients (Table 1). Patients were recruited in this prospective, multicenter study between 12/2018 and 4/2019 at the International Center for Lysosomal Disorders, University Medical Center Hamburg-Eppendorf, and MRI examinations were performed at the Center for MR-Neurography North affiliated to the Department of Neuroradiology, Heidelberg University.

Twenty-six healthy controls (13 males, 13 females, mean age 39.54 years, range 25–73 years) were prospectively enrolled as a control group for DRG volume measurement. Moreover, 32 controls are part of a previously published prospective study (16 males, 16 females, mean age 44.7 years, range 18–86 years) [19] and served as controls for the assessment of DRG permeability. Inclusion criteria for healthy controls were: ≥18 years, no medical history suspicious for FD, absence of neuropathic pain or other sources of pain, diabetes mellitus, alcoholism, any malignant or infectious illness as risk factors for polyneuropathy. Exclusion criteria were any contraindications for MRI.

Moreover, another 21 patients carrying classical Fabry mutations were part of previously published prospective studies (mean age 42.65 years, range 22–59 years) and served as further controls for the comparison of DRG volume and permeability values [6,7].

### 2.2. Imaging Protocol

Examinations were conducted on a 3 Tesla Magnetic Resonance scanner (Magnetom SKYRA, Magnetom VERIO and Magnetom TIM TRIO, Siemens Healthineers, Erlangen, Germany). A 15-channel receive/transmit spine coil, an 8-channel receive body flex coil (Siemens) and a 15-channel receive/transmit extremity coil (Invivo, Gainesville, FL) were used. All patients and healthy controls underwent MRN including:

1. A three-dimensional (3D) T2-weighted (T2w) sampling perfection with application-optimized contrasts using different flip-angle evolution (SPACE) short-tau-inversion-recovery (STIR) sequence of the lumbosacral plexus: repetition time/echo time 3.000/208 ms, inversion time 210 ms, effective echo time 68 ms, matrix size 320 × 320 × 104, field of view 305 × 305 mm^2^, slice thickness 1.0 mm, no gap, voxel size 1.0 × 1.0 × 1.0 mm^3^, and acquisition time 8:35 min, imaging the lumbosacral spine from the second lumbar vertebra to the coccyx.
2. A T1-weighted, dynamic contrast enhanced (DCE) volumetric-interpolated breath-hold examination (VIBE) sequence (repetition time/echo time 3.3/1.11 ms, flip angle 158, 24 slices, resolution 1.3 × 1.3 × 3.0 mm^3^) covering the pelvis from the upper plate of the fifth lumbar vertebra to the second sacral vertebra. Contrast agent (Dotarem, Guerbert, France) was administered intravenously at a concentration of 0.1 mmol/kg with a flow rate of 3.5 mL/s by automated injection. A total of 24 frames were recorded with a rate of 7.46 s per frame.

### 2.3. Imaging Analysis

Imaging analyses were performed as published previously [6,7,19]. Briefly, assessment of DRG volume was performed in the T2 weighted, 3D sequence of the lumbosacral plexus by measuring the largest diameter of the L3 to S2 DRG in coronal, sagittal, and axial reformations with Osirix (Pixmeo, Bernex, Switzerland). Volumes were calculated in analogy to the mathematical equation for the volume of an ellipsoid: volume in mm^3^ = (horizontal × sagittal × coronal diameter)/2. DRG permeability (K^trans^) of the right and left DRG L5, S1, and the right spinal nerve L5 were performed by drawing a free-hand region of interest (ROI) along the DRG contour within the anatomical T1 VIBE map and transferred to the calculated K^trans^-map, using the commercially available software plug-in IB DCE 1.2 (Imaging Biometrics, Elm Grove, WI, USA) to Osirix.

### 2.4. Skin Biopsies

To assess intraepidermal nerve fiber densities (IENFD), skin punch biopsies were performed in four patients, using a standardized procedure [20,21]. Eight mm punch biopsies were taken 10 cm above the lateral malleolus of the right foot. Skin samples were immediately transferred into Zamboni’s solution and taken directly to the institute of neuropathology for further analysis. Briefly, the biopsy specimen was fixed in a cryoprotective solution, cut in 50 µm sections and immunostained using a polyclonal panaxonal marker (PGP 9.5 antibody, Dako Z5116). IENFD was determined and counted under a light microscope at high magnification (40×) by an experienced neuropathologist (M.G.) to obtain nerve fiber density (fibers/mm^2^).

### 2.5. Normative Reference Values and Statistical Analysis

IENFD was individually assessed in each patient and compared to normative median and lower 5% percentile values for female sex and the corresponding decade [20]. Additionally, individual age-adapted normative reference values for the lower 5% percentile were calculated using a polynominal curve-fitting of order 2, described by the following equation: Age-adapted lower 5% percentile = (0.0007 × age^2^) − (0.2 × age) +13. Subsequently, percentage difference between age-adapted lower 5% percentile and patients IENFDs was calculated individually as following: IENFD difference = 100 × (Patient IENFD − Age-adapted lower 5% percentile)/(Age-adapted lower 5% percentile), (Table 1).

### 2.6. Statistical Analysis

Statistical analyses were performed with GraphPad Prism 7.0 (GraphPad Software, La Jolla, USA). Mean values for DRG volumes L3 to S2, vascular permeability of DRGs L5 to S1, and spinal nerve L5 were calculated and tested for statistical significance using one-way Analysis of Variance (ANOVA) with Bonferroni correction; *p*-values of < 0.05 were considered significant. All results are documented as mean values ± standard deviation.

## 3. Results

Quantitative analyses of DRG morphology and perfusion were assessed for a total of five patients carrying the Fabry related GLA-gene variant p.A143T and compared to 21 patients carrying a classical Fabry mutation and 26 healthy controls.

**Figure 1:**
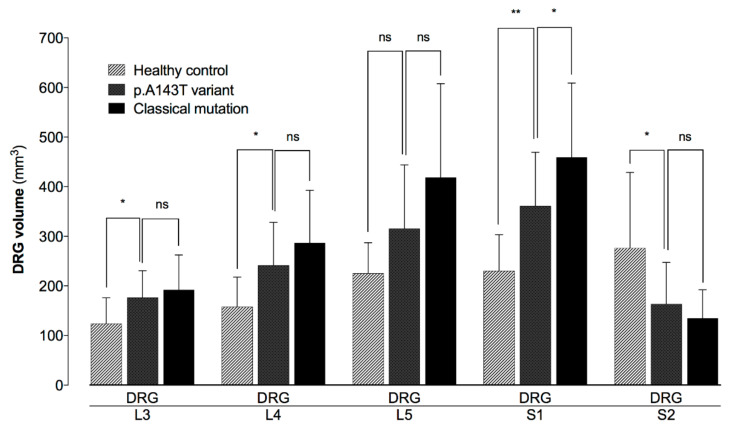
Quantitative assessment of dorsal root ganglia (DRG) volumes L3 trough S2. Mean values of DRG volumes L3 through S2 were calculated level-wise for patients carrying the *GLA*-gene variant p.A143T, patients with a classical Fabry mutation and healthy controls. Compared to healthy controls, p.A143T carriers showed increased DRG volumes for the level L3, L4, S1, and S2, but less increased compared to patients carrying a classical Fabry mutation (ns non-significant, * *p* < 0.05, ** *p* < 0.01).

Compared to healthy controls, DRG volumes of p.A143T patients were symmetrically increased by 30% for the DRG level L3 (176.31 ± 54.33 mm^3^ vs. 124.11 ± 51.72 mm^3^, Bonferroni-adjusted *p* < 0.05), 35% for L4 (241.48 ± 86.70 mm^3^ vs. 157.97 ± 59.73 mm^3^, Bonferroni-adjusted *p* < 0.05), 29% for L5 (315.58 ± 128.41 mm^3^ vs. 225.22 ± 61.64 mm^3^, Bonferroni-adjusted *p* = 0.15), 36% for S1 (361.11 ± 108.27 mm^3^ vs. 230.34 ± 72.84 mm^3^, Bonferroni-adjusted *p* < 0.01), and 18% for S2 (163.73 ± 83.60 mm^3^ vs. 134.74 ± 57.50 mm^3^, Bonferroni-adjusted *p* < 0.05) (Figure 1) (Table 1).

Compared to patients with a classical Fabry mutation, DRG volumes of p.A143T carriers were decreased by 8% for the DRG level L3 (176.31 ± 54.33 mm^3^ vs. 192.03 ± 70.28 mm^3^, Bonferroni-adjusted *p* > 0.99), 16% for L4 (241.48 ± 86.70 mm^3^ vs. 286.46 ± 106.18 mm^3^, Bonferroni-adjusted *p* = 0.39), 25% for L5 (315.58 ± 128.41 mm^3^ vs. 418.57 ± 188.91 mm^3^, Bonferroni-adjusted *p* = 0.09), 21% for S1 (361.11 ± 108.27 mm^3^ vs. 459.09 ± 149.81 mm^3^, Bonferroni-adjusted *p* < 0.05), and 41% for S2 (163.73 ± 83.60 mm^3^ vs. 276.12 ± 152.37 mm^3^, Bonferroni-adjusted *p* > 0.99) (Figure 1).

**Figure 2:**
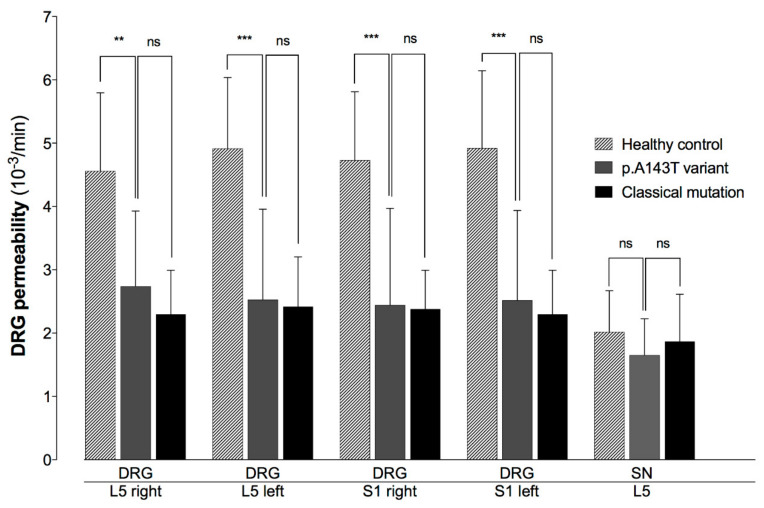
Permeability (K^trans^) of the dorsal root ganglia (DRG) L5, S1 and spinal nerve (SN) L5. K^trans^ as a marker of vascular permeability was assessed in p.A143T patients in comparison to patients with a classical Fabry mutation and healthy controls. Compared to healthy controls, p.A143T carriers showed decreased permeability for all DRGs, but non-significant less decreased compared to patients carrying a classical Fabry mutation. Spinal nerve L5, as internal reference, showed no differences between p.A143T carriers, patients with a classical Fabry mutation, and healthy controls. (ns non-significant, ** *p* < 0.01, *** *p* < 0.001).

Compared to healthy controls, DRG perfusion was decreased in p.A143T patients by 40% for the right DRG L5 (2.74 ± 1.19 10^−3^/min vs. 4.56 ± 1.23 10^−3^/min, Bonferroni-adjusted *p* < 0.01), by 49% for left L5 (2.53 ± 1.43 10^−3^/min vs. 4.92 ± 1.12 10^−3^/min, Bonferroni-adjusted *p* < 0.001), by 48% for right S1 (2.44 ± 1.52 10^−3^/min vs. 4.73 ± 1.08 10^−3^/min, Bonferroni-adjusted *p* < 0.001), and by 49% for left S1 (2.52 ± 1.42 10^−3^/min vs. 4.92 ± 1.22 10^−3^/min, Bonferroni-adjusted *p* < 0.001). Permeability of the spinal nerve L5 showed no change between p.A143T patients and healthy controls (K^trans^ SN: 1.65 ± 0.57 10^−3^/min vs. 2.02 ± 0.65 10^−3^/min, Bonferroni-adjusted *p* = 0.77) (Figure 2) (Table 1).

Compared to patients carrying the GLA-gen variant p.A143T, patients with a classical Fabry mutation showed a decreased vascular permeability by 16% for the right DRG L5 (2.74 ± 1.19 10^−3^/min vs. 2.30 ± 0.69 10^−3^/min, Bonferroni-adjusted *p* > 0.99), by 4% for the left L5 (2.53 ± 1.43 10^−3^/min vs. 2.42 ± 0.79 10^−3^/min, Bonferroni-adjusted *p* > 0.99), by 3% for the right S1 (2.44 ± 1.52 10^−3^/min vs. 2.38 ± 0.61 10^−3^/min, Bonferroni-adjusted *p* > 0.99), and by 9% for the left S1 (2.52 ± 1.42 10^−3^/min vs. 2.30 ± 0.69 10^−3^/min, Bonferroni-adjusted *p* > 0.99): Spinal nerve permeability, however, was increased in patients carrying a classical Fabry mutation by 13% (K^trans^ SN: 1.65 ± 0.57 10^−3^/min vs. 1.87 ± 0.75 10^−3^/min, Bonferroni-adjusted *p* > 0.99) (Figure 2).

IENFD was assessed in four out of five enrolled patients and compared to normative values adapted to sex, decade, and age (Table 1). Compared to median normative reference values per decade, three out of four p.A143T carriers showed a reduced IENFD by 77% in patient 1 (1.33 vs. 5.90 nerve fibers/mm^2^), by 68% in patient 2 (2.80 vs. 8.70 nerve fibers/mm^2^), and by 85% in patient 4 (1.60 vs. 10.70 nerve fibers/mm^2^) (Table 1).

Compared to 5% lower normative reference values per decade, IENFD was reduced by 34% in patient 1 (1.33 vs. 2.00 nerve fibers/mm^2^), by 32% in patient 2 (2.80 vs. 4.10 nerve fibers/mm^2^), and by 74% in patient 4 (1.60 vs. 6.10 nerve fibers/mm^2^) (Table 1).

Compared to 5% lower normative reference values per age, IENFD was reduced by 43% in patient 1 (1.33 vs. 2.30 nerve fibers/mm^2^), by 23% in patient 2 (2.80 vs. 3.60 nerve fibers/mm^2^), and by 76% in patient 4 (1.60 vs. 6.60 nerve fibers/mm^2^) (Table 1).

In patient 5, IENFD was 7.5 fibers/mm^2^, which is in the normal range compared to median normative reference values per decade (7.1 fibers/mm^2^), to 5% lower normative reference values per decade (3.2 fibers/mm^2^), and to 5% lower normative reference values per age (4.0 fibers/mm^2^).

## 4. Discussion

By applying morphological and functional MRN and determination of IENFD, this study examined the individual pathological significance of the disease-modifying *GLA*-variant p.A143T with regard to the PNS. As a principal finding, we here report a distinct increase of DRG volume, a decrease of DRG perfusion and a reduction of IENFD in patients carrying p.A143T. Overall, these morphological and functional alterations share features which we previously described for male and female patients with a disease-causing Fabry mutation as well as for female patients with the disease-modifying variant p.D313Y [6,7,17].

While there is a general agreement on diagnostic criteria and Fabry specific treatment of multi-symptomatic patients with a classical, disease-causing variant, the therapeutic strategies in oligo-symptomatic patients with disease-modifying, late-onset variants like p.A143T represent a major challenge [10,22,23,24,25]. As classical Fabry mutations are commonly associated with a concomitant occurrence of early strokes, left ventricular hypertrophy, myocardial infarction, cardiac arrhythmia, as well as progressive renal impairment, patients with the p.A143Y variant are generally asymptomatic in young age and late-onset symptoms are generally restricted to a single organ system. As the majority of those patients show normal or only slightly reduced *GLA*-activity with normal lyso-Gb levels, it is regularly questioned if a single organ manifestation like renal impairment or left ventricular hypertrophy has to be attributed causally to the Fabry-related variant or should be seen as a non-relevant, coincidental finding [10,23,24]. The finding that no Gb3 deposits could be found in renal or cardiac tissue of affected patients aggravated this question [24,26]. Thus, up to date, the pathophysiological significance of certain *GLA*-variants has to be addressed individually in a multidisciplinary approach for every single patient, as this has important consequences for further diagnostic monitoring and Fabry specific therapy.

A previous study that retrospectively analyzed the medical history of p.A143T patients in comparison to carriers of a classical Fabry mutation found less severe Fabry related symptoms in p.A143T patients, while cardiac or renal involvement as typical Fabry related complications were completely absent [23]. In this regard, *GLA*-activity in p.A143T patients was normal or only slightly decreased predominantly in men, but lyso-Gb3 levels were mostly within normal limits. However, some of those patients exhibited an increased incidence of cardiovascular events like cryptogenic strokes and transient ischemic attacks. This subgroup of patients also revealed slightly decreased *GLA*-activity, and/or increased lyso-Gb3 levels. This leads to the assumption that the p.A143T variant might represent a phenotype that is predominantly restricted to the nervous system [23,27,28]. In our p.A143T cohort, only patient number 3 showed cerebral white matter lesion in cerebral MRI, but those could also be attributed to a long-term hypertension. Additional cerebral MRIs in patient 2 and 5 found no evidence for cerebral white matter lesion or other CNS manifestations. Moreover, none of our patients had a transient ischemic attack or a major stroke in his past medical history. Interestingly, cardiac and renal impairment was much more common in our cohort. However, it remains speculative if these are coincidental findings or could be attributed to the underlying disease.

In FD, the central nervous system (CNS) and PNS and its vascular supply seems to be highly sensitive to a reduced *GLA*-activity and or elevated lyso-Gb3 levels [29,30,31]. Thus, neuropathic pain and pain crises in terms of a SFN are common and early symptoms that occur usually within the first two decades of life [4]. Investigations of the PNS might therefore serve as a helpful tool in the determination of the pathophysiological significance of disease-modifying variants of unknown significance. Correlation analyses of morphological and functional PNS alterations with the occurrence of neuropathic pain as one of the earliest symptoms in FD might offer new insights into the pathophysiology of FD. Moreover, these tools may help to identify patients at risk for cardiovascular events and to promote early diagnosis of major system involvement and therapeutic decisions on an individual basis.

In a previous study, we investigated patients with the disease-modifying *GLA*-variant p.D313Y by morphological and functional MRN. Thereby, we demonstrated that these patients share pathological alterations of the DRG and peripheral nerve that are commonly seen in multi-symptomatic Fabry patients with classical mutations [17]. However, p.D131Y patients primarily showed distinct enlarged DRGs, while DRG perfusion remained within normal limits. In our p.A143T cohort, all patients revealed severely decreased DRG perfusion to a similar extend compared to patients with classical mutations. This possibly could be a hint that the p.A143Y variant might affect vascular permeability within the DRG to a similar degree as patients with classical FD.

Moreover, in an ongoing study, we diagnosed SFN in 7 out 9 p.D313Y patients by obtaining skin biopsies. Thus, the affection of the PNS seems to be not only a common and early feature of classical Fabry mutations, but also of disease-modifying variants like p.D313Y or p.A143T. To gain a reliable conclusion of the pathological significance of p.A143T in general, it would be reasonable to evaluate new diagnostic biomarkers of major organ involvement, as the ones presented here, in larger multicenter studies, that contain also asymptomatic p.A143T carriers.

Considering that at least some p.A143T patients share DRG volume and perfusion characteristics commonly seen in classical FD, the findings of this study raise the question of whether these patients should be monitored closely for PNS long-term complications and Fabry specific treatment might be considered in at least those patients showing Fabry related PNS alterations and symptoms. Diagnostic tools such as peripheral nerve imaging and determination of IENFD might help to critically evaluate the relation of PNS involvement on an individual basis, as this could have important consequences for FD specific therapies [32,33].

A limitation of this study is the relatively small sample size. Thus, the transfer of the results of this study to p.A143T carriers in general should be made very carefully and individually in order to the patient’s medical history and symptoms. Second, the p.A143T population is of slightly elevated age compared to healthy controls and patients with a classical Fabry mutation. Although previous studies revealed no significant correlation of DRG volume and perfusion with age, we cannot exclude any effect of demographic determinants to DRG characteristics with absolute certainty.

## 5. Conclusions

Oligo-symptomatic patients with the disease modifying *GLA*-variant p.A143T show alterations of the PNS with enlarged DRGs, while DRG perfusion and IENFD is reduced. Overall, these alterations resemble characteristics that are seen in patients with a classical disease-causing mutation as well as in patients with the disease-modifying variant p.D313Y. MRN and skin biopsy may help to detect early affections of the PNS as a major organ system in patients with disease modifying *GLA*-variants on an individual basis.

## Figures and Tables

**Table 1:** Patient demographics and clinical data Mainz Severity Score Index (MSSI). ^a^ Reference values: Lyso-Gb3 (plasma) <2.2 ng/mL. ^b^ compared to <5% norm/age, ^c^ compared to sex-matched controls.

Patient	Age (at Diagnosis)	Neurological Symptoms/Clinical Features	MSSI	Lyso-Gb3 Level (ng/mL) ^a^	IENFD	<5% Norm/Decade	<5% Norm/Age	IENFD Change ^b^	DRG Volume (L3-S2) ^c^	DRG Permeability (L5-S1) ^c^
1	71 (61)	Hypohidrosis, angiokeratosis, tinnitus, cardiomyopathy (<15 mm), valve insufficiency, hypertension, proteinuria	18	1.3	1.33	2.0	2.3	−43%	+29%	−63%
2	59 (56)	Myocardial infarction, occasional acroparesthesia, migraine	3	1.1	2.8	4.1	3.6	−23%	+54%	−35%
3	69 (62)	Valve insufficiency, hypertonia, cerebral white matter lesions, tinnitus	3	1.1	n.a	n.a.	n.a.	n.a.	+55%	−77%
4	37 (34)	Edema, hyperhidrosis, vertigo	4	0.8	1.6	6.1	6.6	−6%	+17%	−12%
5	56 (52)	Myocarditis, valve insufficiency, renal insufficiency (stadium II), pruritus, acroparesthesia, depression, hypertonia, NYHA I	5	1.1	7.5	3.2	4.0	+88%	+43%	−49%
